# Supplementary material for: Characteristic of Concurrent Uterine Lipoleiomyoma and Hemangioma by Algorithm of Candidate Biomarkers for Uterine Mesenchymal Tumor
Source: Diagnostics (Basel). 2022 Oct 12;12(10):2468. doi: 10.3390/diagnostics12102468 (PMC9600651; doi:10.3390/diagnostics12102468)
Supplement: Supplementary file 1 [file diagnostics-12-02468-s001.zip › Supplementary Figures S1,2.pdf]

Supplementary Information

Supplementary Materials for

Title: Characteristic of concurrent Uterine lipoleiomyoma and hemangioma by algorithm of candidate biomarkers for uterine mesenchymal tumor

Shoko Nishikawa, Takuma Hayashi, Yasuaki Amano, Nobuo Yaegashi, Kaoru Abiko, Ikuo Konishi

1. Clinical situation history of a patient with lipoleiomyoma and hemangioma

March 2014: The patient was seen at a nearby general hospital due to menstrual irregularities. Multiple subserous uterine tumors of approximately 3 cm in size were observed by MRI imaging, and no anemia was observed.
May 2016: In May 2016, at the time of clinical examination, the uterine fibroid, which was 3 cm in size, had grown to 4.5 cm. Follow-up clinical observation was continued.
May 2019: Since May 2019, irregular menstruation (cycle: 5 days/6 months) had occurred.
June 2021: The patient sought consult at a nearby general hospital because of abnormal vaginal bleeding. MRI examination showed an increase in size of the uterine leiomyoma.
June 20, 2021: Since the patient requested laparoscopic surgery for the uterine leiomyoma, the patient was referred to our medical institution from the nearby general hospital by the attending physician.
June 20, 2021: The patient has not visited our medical facility for about a year.
April 19, 2022: The patient presented to our medical facility for abnormal vaginal bleeding.
Blood pressure: 128/79   Heart rate: 67
Results:
MRI results An intramuscular mass measuring 13 × 13 × 9.5 cm in diameter was found in the anterior wall of the uterus. The boundary between normal tissue and tumor was clear.
MRI T1 imaging and MRI T2 imaging examinations were not suppressed in high, FatSat. There were no findings suggesting malignancy from the results of the MRI.
The results of the MRI imaging examination revealed findings suggestive of degenerative myoma.
The endometrium is slightly compressed and stretched.

Vaginal discharge: small amount
Cervix: nothing particular
C.ut: newborn head size, no left/right mobility

Transvaginal ultrasonography
Uterus: endometrium is thin; a well-demarcated highly echoic tumor was observed.
Transabdominal ultrasound diameter: 115 × 92 mm
Ovary: bilateral not detected
Ascites (negative)

Surgery date: May 10, 2022
Preoperative diagnosis: Leiomyoma
Surgical procedure: Total abdominal hysterectomy, bilateral salpingo-oophorectomy
Total weight of the uterus and both fallopian tubes: 1913 g
Surgical description: Supine position. Surgery was performed under general anesthesia.

2. Clinical situation history of a patient with concurrent uterine leiomyoma and leiomyosarcoma

Regarding the photographs of tissues stained by immunohistochemical staining using uterine leiomyoma and uterine leiomyosarcoma tumor tissue in the section of result, this uterine tumor was excised from a patient suspected of developing uterine leiomyosarcoma by our medical team. In many patients, uterine leiomyosarcoma is co-occurred with uterine leiomyoma. So, uterine leiomyoma and uterine leiomyosarcoma tumor tissue were surgically excised from the same patient.

Age: 70s, **Sex:** Female

April 2019: we medical staff performed surgical treatment for patients suspected of developing uterine leiomyosarcoma: abdominal simple total hysterectomy, bilateral appendagectomy, reticulometry of the reticulum, and mesentero-disseminated lesion resection.

May-October 2019: Combination therapy with DTX and GEM was started (6 cycles in total)
October 2021 PET-CT scan examination revealed recurrence of the tumor in the pelvis
Treatment with administration to Pazopanib was initiated

Jan 2022: CT scan examination rate the therapeutic efficacy of Pazopanib as PD

February 2022: Treatment with administration to DXR was started

July 2022: Currently: the eighth cycle has ended.
By CT, the therapeutic effect of DXR was evaluated as SD.

Histopathological diagnosis: Concurrence of uterine leiomyoma and uterine leiomyosarcoma is recognized.

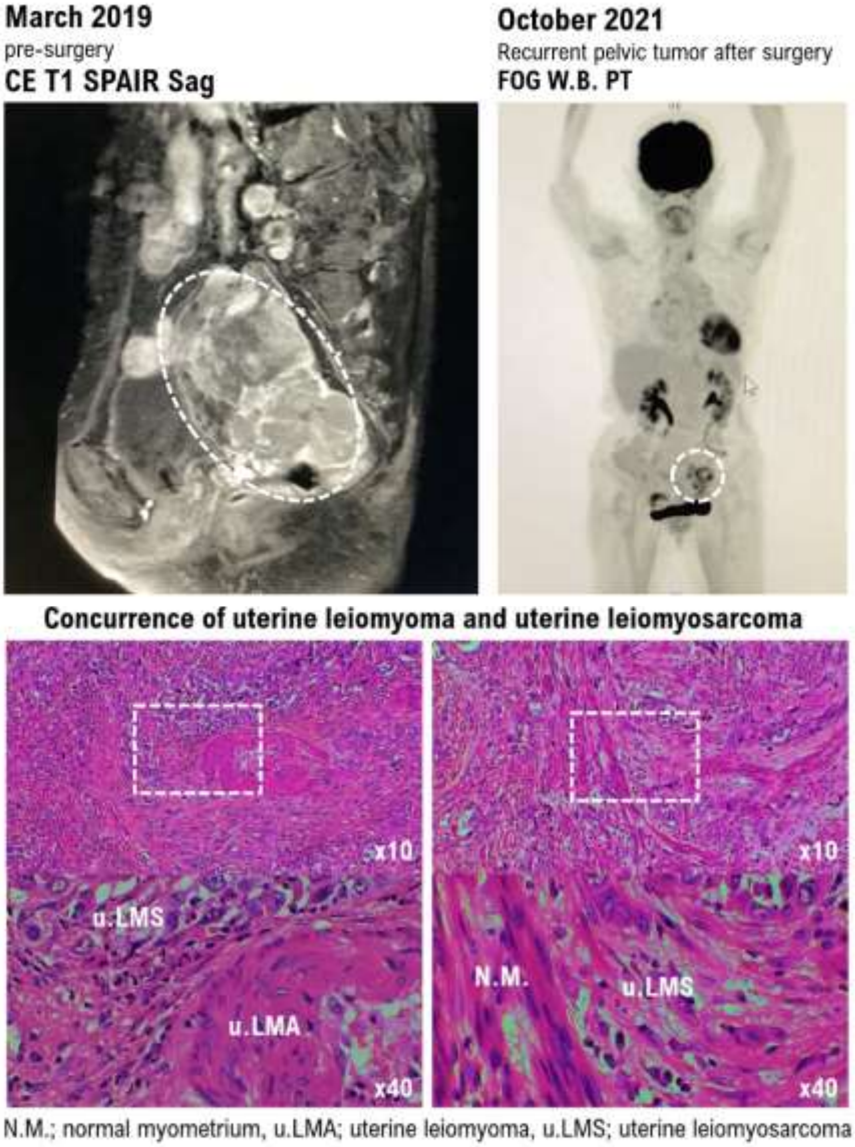

Appendix.
Patient with concurrent uterine leiomyoma and leiomyosarcoma

Left Panel: Image obtained by CE T1 APAIR Sag examination
Right Panel: Image obtained by FOG W.B. PT examination
The white dotted circle indicates the uterine tumor.

Lower Panel: Images obtained by Hematoxylin-Eosin Staining examination

2. Materials and methods

1. Tissue collection

A total of 101 patients between 32 and 83 years of age and diagnosed with smooth muscle tumors of the uterus were selected from the pathological files. Serial sections were cut from at least two tissue blocks from each patient for hematoxylin and eosin staining and immunostaining. After obtaining written consent from each patient, all tissues were used with the approval of the Ethical Committee of Shinshu University. The pathological diagnosis of uterine smooth muscle tumors was confirmed using the established criteria (Hendrickson and Kempson, 1995) with some modification. Briefly, usual leiomyoma (usual LMA) was defined as a tumor showing typical histological features with a mitotic index (MI) [obtained by counting the total number of mitotic figures (MFs) in 10 high-power fields (HPFs)] of <5 MFs per 10 HPFs. Cellular leiomyoma (cellular LMA) was defined as a tumor with significantly increased cellularity (>2000 myoma cells/HPF) and an MI<5, but without cytologic atypia. Bizarre leiomyoma (BL) was defined as a tumor either with diffuse nuclear atypia and an MI <2 or with focal nuclear atypia and an MI <5 without coagulative tumor cell necrosis. A tumor of uncertain malignant potential was defined as a tumor with no mild atypia and an MI <10 but with coagulative tumor cell necrosis. Leiomyosarcoma (LMS) was diagnosed in the presence of an MI >10 with either diffuse cytologic atypia, coagulative tumor cell necrosis, or both. Of the 105 smooth muscle tumors, 52 were diagnosed as LMA, 3 were BL, 2 were intravenous leiomyomatosis, 58 were uterine LMS, 1 was uterine LANT-like tumor, 2 were uterine lipoleiomyoma and 2 were uterine rhabdomyosarcoma. Of the 58 LMS, histologically, 48 were of the spindle cell type and 10 were of the epithelioid type. The clinical stage of the LMS patients was stage I in 11 cases, stage II or III in 31 cases, and stage IV in 16 cases. The protein expression studies with cervical epithelium and carcinoma tissues were performed using tissue array (Uterus cancer tissues, AccuMax Array, Seoul, Korea). Details about tissue sections are indicated in the manufacture's information (AccuMax Array).

2. Immunohistochemistry (IHC)

IHC staining for caveolin 1, cyclin B, cyclin E1, large multifunctional peptidase 2/β1i (LMP2/β1i), Ki-67, desmin, and myogenin was performed using serial human uterine mesenchymal tumor sections obtained from patients with uterine mesenchymal tumor (Supplementary Material 1). A monoclonal antibody against cyclin E1 (CCNE1/2460) was purchased from Abcam (Cambridge Biomedical Campus, Cambridge, UK) and a monoclonal antibody against Ki-67 (clone MIB-1) was purchased from Dako Denmark A/S (DK-2600 Glostrup, Denmark). Monoclonal antibodies against desmin (clone RM234) and myogenin (clone MGN185) were purchased from GeneTex, Inc. (Irvine, CA, USA). The monoclonal antibody against caveolin 1, cyclin B1, and LMP2/b1i were purchased from Santa Cruz Biotechnology Inc. (Santa Cruz, CA, USA). IHC was performed using the avidin–biotin complex method as described previously [8,9,10]. Briefly, one representative 5-mm tissue section was cut from a paraffin-embedded sample of a radical hysterectomy specimen from each patient with a uterine mesenchymal tumor.

The sections were incubated with a biotinylated secondary antibody (Dako, DK-2600 Glostrup, Denmark) followed by the streptavidin complex (Dako). The completed reaction was developed using 3,39-diaminobenzidine and the slides were counterstained with hematoxylin. Normal myometrial tissues in the specimens were used as positive controls. The negative controls consisted of tissue sections incubated with normal rabbit IgG instead of the primary antibody. Shinshu University approved the experiments according to internal guidelines (approval no. M192). The expression of cyclin E and Ki-67/MIB1 was indicated by brown 3,3'-diaminobenzidine (DAB) tetrahydrochloride staining using a standard procedure. Normal rabbit or mouse antiserum was used as a negative control for the primary antibody. The entire brown DAB tetrahydrochloride-stained tissue was scanned using a BZ-X800 digital microscope (Keyence, Osaka, Osaka, Japan). The expression of cyclin E and Ki-67/MIB1 were indicated by brown dots.

IHC staining for CD31 was performed on the excised tissue sections. Briefly, tumor tissue sections were incubated with the appropriate primary antibodies at 4°C overnight. The primary antibodies were a mouse monoclonal antibody to CD31 (1:200). A monoclonal antibody for CD31 (clone JC/70A) was purchased from GeneTex, Inc. (Irvine, CA, USA). Following incubation with an Alexa Fluor® 488-conjugated anti-mouse IgG or Alexa Fluor® 546-conjugated anti-rabbit IgG secondary antibody (1:200; Invitrogen), the sections were washed, cover-slipped with mounting medium and 40,6-diamidino-2-phenylindole (Vectashield; Vector Laboratories, Burlingame, CA, USA), and visualized by confocal microscopy (Leica TCS SP8, Wetzlar, Germany) according to the manufacturer's instructions. Normal rabbit or mouse antiserum was used as a negative control for the primary antibody. The experiments with human tissues were conducted at the National Hospital Organization Kyoto Medical Center in accordance with institutional guidelines (approval no. NHO H31-02).

3. Ethical approval and consent to participate

The Central Ethics Review Board of the National Hospital Organization Headquarters in Japan (Tokyo, Japan) and Shinshu University (Nagano, Japan) reviewed and approved this study. Ethical approval was obtained on August 17, 2019 with the code NHO H31-02. The authors attended educational lectures on medical ethics in 2020 and 2021, which were supervised by the Japanese government. The completion numbers for the authors are AP0000151756, AP0000151757, AP0000151769, and AP000351128. Consent to participate was required as this research was considered clinical research. The participants signed an informed consent form during the clinical study briefing and agreed with content of the research. The authors attended a seminar on the ethics of experimental research using small animals on July 02, 2020 and July 20, 2021. They became familiar with the importance and ethics of animal experiments (National Hospital Organization Kyoto Medical Center and Shinshu University School of Medicine). The code number for the ethical approval for experiments involving small animals was KMC R02-0702.

Nishikawa *et al.* S.Figure 1

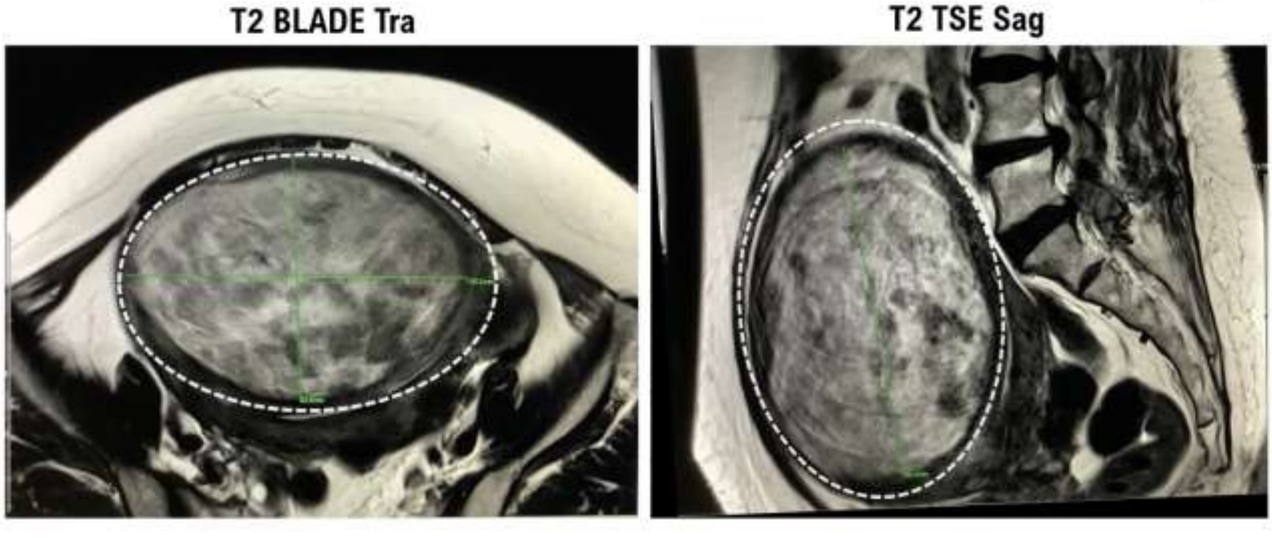

Supplementary Figure S1. Contrast images of magnetic resonance imaging (MRI)
Contrast MRI images, that is, T2 BLADE Tra and T2TES Sag, clearly show the mass of the Our Case (patient with uterine tumor). The white dotted circle indicates the uterine tumor.

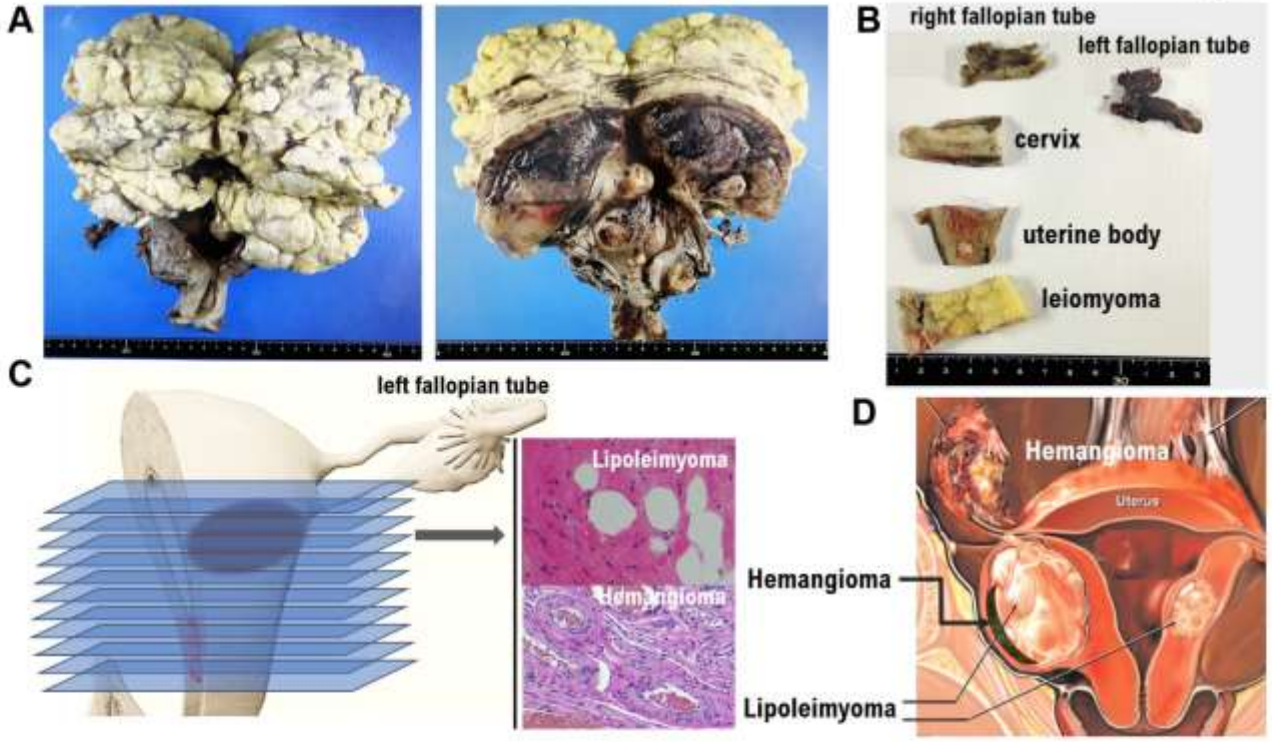

Supplementary Figure S2. Gross and histopathological morphology of the uterine mesenchymal and vascular tumors. A. Gross findings of excised tissue fixed in formalin. The black discolored tissues contain many capillaries. Hemangioma develops and proliferates in these tissues in which a large amount of blood flow is observed. **B.** At the uterine body, gray-white masses are observed (upper masses) which included the leiomyoma tissue. No evidence of benign or malignant tumors is expected in the cervical tissue block. In the fallopian tubes and ovaries on both sides, a dark discolored tissue is observed. Normal fallopian tubes and ovarian tissue have many capillaries, hence the prominent blood flow. Therefore, a black discoloration is seen in the fallopian tubes and ovaries due to this large amount of blood flow. **C.** The cell tissue findings in each block, where the excised tissue was cut into 11 pieces were examined. The results show lipoleiomyoma and hemangioma in the tissue contained in the fourth block from the top. **D.** From the histopathological findings on the 11 block sections of the excised tissue, it is believed that the hemangioma did not grow inside the lipoleiomyoma. Rather, the hemangioma grew in the vicinity of the lipoleiomyoma tissue.
