# Supplementary material for: Characteristic of Concurrent Uterine Lipoleiomyoma and Hemangioma by Algorithm of Candidate Biomarkers for Uterine Mesenchymal Tumor
Source: Diagnostics (Basel). 2022 Oct 12;12(10):2468. doi: 10.3390/diagnostics12102468 (PMC9600651; doi:10.3390/diagnostics12102468)
Supplement: Supplementary file 1 [file diagnostics-12-02468-s001.zip › Supplementary Table S1.pdf]

Tumor type	Atypia	Mitotic activity	Necrosis	Protein expression*													Cliical comments
				Cyt	Des	CAV	SMA	Vim	ER/PR	End	EGF	CyB	CyE	PSMB9	Cal	Ki67	
**Endometrial stromal tumors.**																	
Endometrial stromal nodule	minimal	infrequent	-/inconspicuous	+	-	++	+	+	+++	+	*	+	-	++	++	-	Absence of myometrial infiltration
Endometrial stromal sarcoma	-	infrequent	-/inconspicuous	-/+	-	++	+	+	+++	+	+	+	-	-/+	++	-/+	
Undifferentiated endometrial sarcoma	marked	Frequent (atypical MF)	+	-/+	foc	*	*	-	-	+	+	+	+	-/+	+	+	Lack specific differentiation
**Smooth muscle tumors**																	
Leiomyoma, NOS	-	<5 MF/10HPF	-	foc	+	++	+	*	+++	-/+	-/+	+	-	++	++	-/+	Well-circumscribed
Mitotically active leiomyoma	-	>5 MF/10HPF	-	*	+	++	+	*	+++	-/+	-/+	+	-	++	++	-/+	Pseudocapsul
Cellular leiomyoma	-	infrequent	-	*	+	++	+	*	+++	-/+	-/+	+	-	++	++	-/+	Increased cellularity
Hemorrhagic cellular leiomyoma	-	infrequent	-	*	+	++	+	*	+++	-/+	-/+	+	-	++	++	-/+	Hormone induced changes
Epithelioid leiomyoma	-	<5 MF/10HPF	-	*	+	++	+	*	+++	-/+	-/+	*	-	++	++	-/+	Epithelial-like cells
Myxoid leiomyoma	-	<5 MF/10HPF	-	*	*	*	*	*	*	-/+	-/+	*	+	-/+	+	+	Myxoid material
Atypical leiomyoma	moderate	<10 MF/10HPF	-	-	+	++	+	*	+++	+	-/+	+	+	-/+	-/+	+	Separates tumor cells
Lipoleiomyoma	-	infrequent	-	*	+	++	+	*	+++	*	-/+	*	+	-/+	*	-/+	Scattered adipocytes
STUMP#	-	infrequent	-	*	+	++	+	*	*	*	-/+	*	-/+	-/+	*	-/+	
	-/+	>10 MF/10HPF	+/uncertain	*	+	++	+	*	*	*	-/+	*	-/+	-/+	*	-/+	
	Marked	borderline	-	*	+	++	+	*	*	*	-/+	*	-/+	-/+	*	-/+	
	-	infrequent	+/difficult classify	*	+	++	+	*	*	*	-/+	*	-/+	-/+	*	-/+	
Intravenous leiomyomatosis	moderate	infrequent	-	+	*	++	+	*	*	*	*	-/+	-/+	-	-	+	Infiltrative
Leiomyosarcoma	moderate	>10 MF/10HPF	+	+	+	++	-/+	-	-	+	-/+	++	+++	-	-	++	Infiltrative
Leiomyosarcoma epitheliod variant	moderate	>5 MF/10HPF	+	+	+	++	-/+	-	-	+	-/+	++	+++	-	-	++	Infiltrative, >50% epithelioid cells
Leiomyosarcoma myxoid variant	moderate	Any MF	+	+	+	++	-/+	-	-	+	-/+	++	++	-	-	++	Infiltrative, myxoid extracellular matrix
**Leiomyomatoid tumors**																	
LANT#	absent	frequent	+	-	-	+	+	+	*	*	+	*	++	-	-	-/+	NOTE1

Hemangioma	moderate	infrequent	-														CD31 positive
**Origin is endometrial stromal cells**																	
rhabdomyosarcoma	moderate	>10 MF/10HPF	-	NA	NA	++	NA	NA	NA	NA	NA	+	++	++	NA	++	

*insufficient data or not applicable.

Cyt.; cytokeratin, Des.; Desmin, CAV; caveolin 1, SMA; smooth muscle actin, Vim.; vimentin, ER/PR; estrogen receptor/progesterone receptor, End.; Endoglin; CD105/TGFb receptor (stem cell marker), EGF, EGFR; epidermal growth factor receptor, CyB; cyclin B1, CyE; cyclin E, PSMB9; proteasome beta subunit 9, Cal.; calponin h1, CD56; neural cell adhesion molecule (N-CAM), WT-1; wilms tumor 1, NOS; not otherwise specified, MF; magnification factor, HPF; high power field, Foc.; focal, STUMP; smooth muscle tumors of uncertain malignant potential. Protein expression*, estimated-protein expressions by immunoblot analysis, immunohistochemistry (IHC) and/or RT-PCR (quantitative-PCR), +/-; partial expression, +; expression, ++; medium expression, +++; high expression, -; no evidence of expression, ER/PR(ref.13), PSMB9 (ref.13,14), cyclin E(ref.13,14), calponin h1(ref.15), Ki-67(ref.13,14). STUMP#(ref.15,16). Cyclin E, PSMB9, calponin h1 are potential bio-marker for human uterine mesenchymal tumors. LANT#, leiomyomatoid angiomatous neuroendocrin tumour (LANT) is described as a dimorphic neurosecretory tumor with a leiomyomatous vascular component (ref.17). NOTE1, Low-grade neuroendocrine tumor possibly related to null cell adenoma.

Supplementary Table S1. Classification of human uterine mesenchymal tumors
